# Effectiveness of an Online Group Course for Depression in Adolescents and Young Adults: A Randomized Trial

**DOI:** 10.2196/jmir.2033

**Published:** 2012-06-07

**Authors:** Rianne van der Zanden, Jeannet Kramer, Rob Gerrits, Pim Cuijpers

**Affiliations:** ^1^Centre of Mental Health of Youth and AdolescentsTrimbos InstituteUtrechtNetherlands; ^2^Innovation Centre of Mental Health and TechnologyTrimbos InstituteUtrechtNetherlands; ^3^Dimence Institute of Mental HealthDeventerNetherlands; ^4^Department of Clinical PsychologyEMGO Institute for Health and Care ResearchVrije UniversiteitAmsterdamNetherlands

**Keywords:** eHealth, health promotion, depressive symptoms, anxiety, adolescents, Internet, randomized controlled trial

## Abstract

**Background:**

Depression is a serious mental health problem, whose first onset is usually in adolescence. Online treatment may offer a solution for the current undertreatment of depression in youth. For adults with depressive symptoms, the effectiveness of Internet-based cognitive behavioral therapy has been demonstrated. This study is one of the first randomized controlled trials to investigate the effectiveness online depression treatment for young people with depressive complaints and the first to focus on an online group course.

**Objective:**

To evaluate and discuss the effectiveness of a guided Web-based group course called Grip op Je Dip (Master Your Mood [MYM]), designed for young people aged 16 to 25 years with depressive symptoms, in comparison with a wait-listed control group.

**Methods:**

We randomly assigned 244 young people with depressive symptoms to the online MYM course or to a waiting-list control condition. The primary outcome measure was treatment outcome after 3 months on the Center for Epidemiologic Studies Depression Scale. Secondary outcomes were anxiety (measured by the Hospital Anxiety and Depression Scale) and mastery (Mastery Scale). We studied the maintenance of effects in the MYM group 6 months after baseline. Missing data were imputed.

**Results:**

The MYM group (n = 121) showed significantly greater improvement in depressive symptoms at 3 months than the control group (n = 123) (*t*_187 _= 6.62, *P *< .001), with a large between-group effect size of *d *= 0.94 (95% confidence interval [CI] 0.64–1.23). The MYM group also showed greater improvement in anxiety (*t*_187 _= 3.80, *P *< .001, *d *= 0.49, 95% CI 0.24–0.75) and mastery (*t*_187 _= 3.36, *P *= .001, *d *= 0.44, 95% CI 0.19–0.70). At 12 weeks, 56% (68/121) of the participants in the MYM group and 20% (24/123) in the control group showed reliable and clinically significant change. This between-group difference was significant (χ^2^_1 _= 35.0, *P *< .001) and yielded a number needed to treat of 2.7. Improvements in the MYM group were maintained at 6 months. A limitation is the infeasibility of comparing the 6-month outcomes of the MYM and control groups, as the controls had access to MYM after 3 months.

**Conclusions:**

The online group course MYM was effective in reducing depressive symptoms and anxiety and in increasing mastery in young people. These effects persisted in the MYM group at 6 months.

**Trial Registration:**

Nederlands Trial Register: NTR1694; http://www.trialregister.nl/trialreg/admin/rctview.asp?TC=1694 (Archived by WebCite at http://www.webcitation.org/683SBoeGV)

## Introduction

### Depression Among Adolescents

Depression is a major health problem. Worldwide it is the fourth-ranked disorder in terms of disease burden, and it is expected to carry the highest disease burden in high-income countries by 2030 [1]. The 12-month prevalence of depression is now 5.5% in high-income countries [2]. It is a common condition in adolescents and young adults. One study found a 12-month prevalence of 6.7% among 18- to 25-year-olds [3]. Adolescent depression is associated with serious problems, including poor school performance [4], school absenteeism and dropout [5], problematic relations with parents and peers [6], excessive tobacco and alcohol use [7], and suicidal behavior [8].

Subclinical depression is also common, with an estimated prevalence of 17% to 21% among Dutch adolescents [9,10]. It involves having some depressive symptoms that together do not meet the full *Diagnostic and Statistical Manual of Mental Disorders*, fourth edition criteria for major depression [11]. Subclinical depression is a risk factor for the development of major depression within a year [12,13]. The psychosocial functional impairment of people with subclinical depression is comparable with that in those who have a diagnosis of major depression [14,15]. Beyond the personal suffering involved, both major and subthreshold depression impose significant economic burdens in terms of health care costs and production losses in paid and unpaid work [16,17].

Given the high prevalence rates, serious outcomes, and economic burden, the World Health Organization calls for the development of preventive interventions to reduce the burden of this disorder [18,19]. The first onset is usually in adolescence [20], and it is wise to intervene at an early stage. Yet young people experience many barriers to seeking professional help. They tend to deny or underestimate the problems, fear stigmatization, and question the benefits of help [21]. If they do seek help, they often encounter waiting lists [22,23].

### Internet-Based Interventions for Depression

By offering a solution to the stigmatization problem, Internet-based approaches could help in reaching target groups who might otherwise remain untreated. The Internet provides anonymity and the opportunity to take part in an intervention in the privacy of the home. Another strong benefit is that Internet-based approaches enable a reduction in contact hours between professionals and clients, which could help tackle the problem of waiting lists, shortages of therapists, and rising health care costs. Web-based interventions with professional support have been found effective in treating depression in adults, with results comparable with traditional psychological approaches [24-26].

The present study focused on one specific type of Web-based intervention: a professionally facilitated, cognitive behavioral therapy (CBT) group course designed for young people with symptoms of depression. The perceived advantages of online group sessions as compared with individual approaches are social support and mutual recognition by group members (though they remain anonymous to one another) and the reduction of professional contact hours per participant in comparison with individual treatment [27]. Three earlier studies on online group courses in mental health care [28-30] showed that it is possible, both technically and substantively, to conduct Internet-based group courses for adolescents and adults with mental health problems via the Internet. Two of these three studies showed significant positive outcomes in pre–post measurements, though also reporting relatively high attrition rates—a common problem for Internet interventions [28]. The pilot study by Gerrits et al [29] among 140 adolescents with depressive complaints who received the online CBT group course Master Your Mood (MYM) showed a significant decline in depressive symptoms. And the pilot study by Van der Zanden et al [30] of 48 parents with mental illness who received parenting support in an online group course showed significant improvement in parenting skills and parental sense of competence.

### Lack of Outcome Studies for Youth

Despite the clear benefits of Web-based interventions for depression, there is a lack of outcome studies specifically focusing on adolescents and young adults [31,32]. Only two randomized controlled trials have been conducted on prevention programs for depression and anxiety [33,34]. Calear and colleagues [33] found effects of a universal prevention program for male and female participants for anxiety but found effects for depression only in male participants. Van Voorhees et al [34] showed that a Web-based behavior change program in primary care, in combination with either motivational interviewing or brief advice, was associated with declines in depressed mood and levels of depressive symptoms.

### Objective

We evaluate and discuss the effectiveness of a Web-based group course called Grip op Je Dip (Master Your Mood), designed for young people aged 16 to 25 years with depressive symptoms, in comparison with a wait-listed control group. The primary outcome measure was depression, and secondary outcomes were anxiety and sense of control. Based on the results of the pilot study [29] we expected better outcomes for the course group.

## Methods

### Study Design

We conducted a randomized controlled trial with two parallel groups to examine the effectiveness of the MYM course, comparing the intervention group with a waiting-list control group. Ethical approval was granted by an independent medical ethics committee (CCMO no. NL18984.097.07). The trial is registered (NTR1694), and the study protocol has been published [35].

### Participating Mental Health Agencies

A total of 14 mental health care agencies participated in the project, all working with online course participants from all over the Netherlands. The courses were supervised by professional mental health promotion workers, trained in administering the Mini-International Neuropsychiatric Interview (MINI-Plus) [36,37] and in conducting the course.

### Study Population

The inclusion criteria were the following: age 16 to 25 years, informed consent (including parental consent for those less than 18 years of age), and a Center for Epidemiologic Studies Depression Scale (CES-D) score between 10 and 45. Applicants were excluded on indications of suicidal ideation with intent and plan, as assessed with the MINI-Plus.

### Recruitment Procedure

Participants were recruited from the general population by means of promotional materials in general practitioners’ offices and educational institutions. Banners and links were also placed on mental health-related websites and on websites popular with young people. There were no explicit restrictions on country of origin, but the course language was Dutch and the recruitment took place in Dutch. The minimum requirements for Internet access were a stable Internet connection and a recent browser (minimum: Internet Explorer 6.0). Participants also had to be able to read, write, and chat in Dutch on at least the primary school level. Those interested were referred to the MYM website [38] to complete an online preliminary screening questionnaire and apply for the course. Those with CES-D scores between 10 and 45 then received additional information about the study, an informed consent form (including a parental consent form for 16- and 17-year-olds), and a baseline questionnaire. For course applicants scoring 25 to 45 on the CES-D, a mandatory online chat session followed, in which suicidal ideation was assessed with the MINI-Plus interview. Those for whom suicidal ideation and plan were determined were excluded from the study and advised to see their general practitioner.

**Figure 1:**
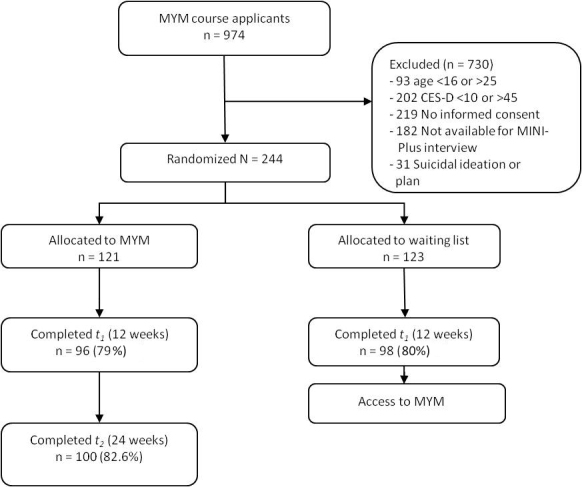
Flow chart of respondent selection. CES-D = Center for Epidemiologic Studies Depression Scale; MINI-Plus: Mini-International Neuropsychiatric Interview; MYM = Master Your Mood.

Eligible applicants were randomly assigned to the intervention group (MYM) or the control group (wait-listed for 14 weeks). Random allocation was automated by a computer program with no interference by course facilitators or researchers. A blocked randomization scheme was used with blocks of two, stratified by depressive symptoms (CES-D scores of 10–24 vs 25–45) and age (younger vs older than 18 years). The outcome of the randomization was generated and made available at the moment the course facilitator indicated that the applicant was eligible for the course. Applicants were informed of their allocation by email and received a tailored referral if they were declined. Participants then received a personal email from their facilitator to inform them of the specific times and dates of the course, the homework assignment for the first session, and a username and password for the chat room. During the trial, participants in both conditions were allowed to seek additional help if they wished. Figure 1 shows a flow chart of respondent selection.

### Conditions: The Intervention

The online MYM course is based on the face-to-face intervention of the same name, which was developed by the Trimbos Institute, the Netherlands Institute of Mental Health and Addiction. That intervention was derived from the Dutch version [39] of the Coping with Depression course [40]. The face-to-face course was adapted to the Internet in a collaborative project involving the Trimbos Institute and three mental health agencies [29,41].

The online MYM group course is a structured form of CBT for depression. At the core of MYM is the cognitive restructuring of thinking patterns. Course participants are encouraged to detect their own unproductive, unrealistic thoughts, and they are then taught to transform these into realistic, helpful thoughts. Performance of pleasant daily activities is also encouraged, and a mood measure is filled in daily to help understand the connection between pleasant activities and mood level.

The course we evaluated took place at fixed times in a secured chat room, which participants entered with their username and password. Anonymity within the group was ensured by a self-chosen nickname. Text messages reminders were sent to participants’ mobile phones one-half hour before each session. The course comprised six sessions of 90 minutes each, each at a set time every week, and home exercises. The sessions were structured around six themes (see Table 1). During the sessions, course material was introduced by the facilitators and displayed in the chat room using text and images. Participants could respond, share experiences, and ask questions. Emoticons could be used to express feelings. Participants and professionals could read through the session transcripts afterward. The course was guided by one or two trained professionals, depending on group size (6 participants was the maximum).

**Table 1:** Outline of the online course Master Your Mood.

Session		Actions
1	Your mood	Learning to understand the relationship between feelings, thoughts, and actions.
2	Your mood and being active	Becoming aware of the influence that activities have on your mood and starting to be more active.
3	Tracing negative thoughts	Becoming aware of negative thoughts and starting to understand what they do to your mood and self-esteem.
4	New ways of thinking	Challenging the negative thoughts and changing them into more positive (but realistic) ones.
5	More action with positive thinking	Combining the new way of thinking with being more active. Becoming more aware of positive things about yourself and others.
6	The future	Making a plan to prevent relapse into depressive moods in the future. Learning the personal signals of a coming depression and knowing how to address that threat. Making a personal plan for the future, to include wishes about schooling, jobs, and relationships and ways to achieve those wishes.

### Conditions: The Waiting List

The wait-listed group did not receive an intervention. They were told by email that they would be invited to participate after the waiting period of 3 months.

### Assessments

Assessments took place before randomization (baseline, *t* *0*), 12 weeks later (*t* *1*), and 12 weeks after that (*t* *2*). Participants received automated emails with invitations to complete the online questionnaires. Subsequent reminders were sent 5 and 10 days after the first email invitation, if necessary. To stimulate response, we offered the participants €10 compensation for completing the *t* *1 *questionnaire and €10 for the *t* *2 *questionnaire, plus an additional €5 bonus (totaling €25) if they completed both.

### Measures

#### Primary Outcome Measure: Depressive Symptoms

The CES-D [42,43] measures the frequency of 20 depressive symptoms over the past week on a 4-point Likert scale. The total score may range from 0 to 60, with higher scores indicating higher levels of depression. Computerized and paper-and-pencil versions of the CES-D correlate at a very high level [44]. The Web-based version of the CES-D has been shown to be a reliable and valid screening instrument in a Dutch adolescent population, with a Cronbach alpha of 0.93 [45]. In our study, the Cronbach alpha was 0.91.

#### Secondary Outcome Measures

##### Anxiety

We used the Anxiety subscale of the Hospital Anxiety and Depression Scale (HADS) to assess anxiety symptoms [46]; the Dutch version of the HADS has been validated [47]. The Cronbach alpha was 0.84 for adults (18+ years) in the general population. The Anxiety subscale consists of 7 items measuring symptoms of anxiety on a 4-point Likert scale, with a score range of 0 to 21 and a higher rating indicating a higher state of anxiety. In our study the Cronbach alpha of this subscale was 0.74.

##### Sense of Control

We used the Dutch version of the 5-item Mastery Scale [48] to assess perceived control. The concept of mastery refers to beliefs about one’s own ability to control one’s environment. Responses are rated on a 5-point Likert scale, with a total score range of 5 to 25; a higher score indicates a greater sense of mastery. The Mastery Scale has good psychometric properties [48]. In our study, the Cronbach alpha was 0.77.

#### Additional Measures

At baseline, we assessed demographic information (sex, age, educational level, and living situation), previous or present professional help for psychological problems, and experience with Web chatting. At 3 months (*t* *1*) and 6 months (*t* *2*), we inquired about subsequent use of professional help and antidepressant medication.

### Analyses

The trial was originally powered to detect a clinical effect of *d *= 0.32 or larger in a 1-sided test with a power of 80% (1 − beta) [29]. Hypotheses were directional, with better outcomes expected for the MYM group. A total of 242 participants were needed for the study, n = 121 per condition (Stata 11.1 syntax: sampsi; StataCorp LP, College Station, TX, USA).

We used *t *tests, chi-square tests, and logistic regression (*P *< .05) to determine whether the randomization had resulted in two comparable groups at baseline and whether any differential loss to follow-up occurred.

The expectation-maximization method was used to impute data missing at *t* *1 *and t_2_. It imputes values by maximum-likelihood estimation using the observed data in an iterative process [49]. These analyses were based on the intention-to-treat principle, including data from all participants, whether or not they received the intervention.

To test course effectiveness, we calculated difference scores between *t* *0 *and *t* *1 *for all outcome variables (positive scores meaning improvement) and compared them across groups in linear regression models, controlling for data clustering (some participants attended the same course sessions). Clustering would violate the assumption of independence of observations and might affect standard errors and *P *values. To adjust for clustering, we obtained robust standard errors and *P *values using the first-order Taylor series linearization method, as implemented in Stata. At *t* *2 *(24 weeks), we studied effect maintenance in the MYM group only; we made no between-group comparisons, as the control group had since been given access to the course.

The sizes of intervention effects were estimated using Cohen *d *[50]. Effect sizes were first calculated for each condition separately, (*t*_0 _− *t*_1_)/SD_t0_, and then the differential effect size was calculated by subtracting the control group effect from that of the MYM group. A difference of *d *= 0.5 would indicate that the experimental group mean was half a standard deviation greater than the control group mean. For Cohen *d*, an effect size of 0.2 to 0.3 may be regarded as a small effect, around 0.5 as a medium effect, and 0.8 to infinity as a large effect.

The proportion of participants showing reliable and clinically significant improvement [51] was determined in terms of an improvement of 5 points on the CES-D in combination with a score lower than 22 on the CES-D (cut-off based on Cuijpers et al [45]). Subsequently, the number needed to treat was calculated as 1/risk difference. The number needed to treat indicates here how many young people with depressive symptoms would need to take the MYM course in order to generate a health gain in 1 person over 12 weeks.

We determined effect maintenance in the MYM group on the basis of the results at 24 weeks (*t* *2*). Paired *t *tests were used to identify significant changes in outcomes between baseline, 12 weeks (*t* *1*), and 24 weeks in the intention-to-treat sample.

## Results

### Participants

Recruitment took place from May 20, 2008 to March 6, 2010. Figure 1 shows the flow of participants through the trial. Of the 974 people who applied for the online MYM course, 244 (25.1%) were included in the study. Reasons for noninclusion were lack of informed consent (219/730, 30.0%), CES-D depression score outside the 10–45 range (202/730, 27.7%), no-show at the MINI-Plus interview (182/730, 24.9%), and age outside the 16–25 range (93/730, 13%). Additional exclusions were made for suicidal ideation (31/730, 4%) and other reasons (3/730, 0%). The 244 selected participants were randomly assigned to one of the two conditions: the online MYM course (n = 121) and the waiting-list control group (n = 123). Table 2 shows baseline demographic and psychosocial characteristics of the 244 participants. Professional help was provided in most cases by a psychologist or other professional from a mental health agency. No significant differences were found on any of these variables between the experimental group and control group (*P *< .05), indicating that the randomization was successful.

**Table 2:** Baseline characteristics of the 244 participants.

Characteristic		Experimental group (n = 121)	Control group (n = 123)	All (N = 244)	Statistics
Female gender, n (%)		101 (83.5%)	105 (85.4%)	206 (84.4%)	χ^2^_1 _= 0.2, *P *= .68
Age (years), mean (SD)		20.8 (2.2)	21.0 (2.3)	20.9 (2.2)	*t*_242 _= 0.6, *P * *= *.53
**Age range (years), n (%)**					χ^2^_2 _=0.2, *P * *= *.92
	16–17	5 (4%)	4 (3%)	9 (4%)	
	18–21	66 (55%)	69 (56%)	135 (55.3%)	
	22–25	50 (41%)	50 (41%)	100 (41.0%)	
**Education level, n (%)** ^a^					χ^2^_2 _=0.6, *P *= .73
	Low	10 (8%)	10 (8%)	20 (8%)	
	Middle	50 (41%)	45 (37%)	95 (39%)	
	High	61 (50%)	68 (55%)	129 (52.8%)	
**Daily activities, n (%)**					χ^2^_2 _=1.9, *P *= .38
	Study	83 (69%)	85 (69%)	168 (69%)	
	Paid employment	32 (26%)	27 (22%)	59 (24%)	
	Other	6 (5%)	11 (9%)	17 (7%)	
**Living situation, n (%)**					χ^2^_3 _= 3.5, *P *= .32
	With parents	56 (46%)	59 (48%)	115 (47.1%)	
	With partner	13 (11%)	18 (15%)	31 (13%)	
	Alone	26 (22%)	16 (13%)	42 (17%)	
	With others	26 (22%)	30 (24%)	56 (23%)	
Experience in Web chat, n (%)		63 (52%)	64 (52%)	127 (52.0%)	χ^2^_1 _= 0.0, *P *= .99
**Professional help, n (%)**					
	Prior	75 (62%)	76 (62%)	151 (61.9%)	χ^2^_1 _= 0.0, *P *= .98
	Current at baseline	36 (30%)	39 (32%)	77 (32%)	χ^2^_1 _= 0.1, *P *= .74
**Test scores, mean (SD)**					
	CES-D depression score^b^	32.5 (8.4)	32.3 (8.2)	32.3 (8.3)	*t*_242 _= 0.28, *P *= .77
	HADS Anxiety^c^	11.2 (3.6)	11.8 (3.7)	11.5 (3.6)	*t*_242 _= 1.27, *P *= .21
	Mastery^d^	12.8 (3.4)	12.8 (3.6)	12.8 (3.5)	*t*_242 _=0.17, *P *= .86

^a ^Combination of highest completed or present education: low = primary or lower secondary school or less; middle = intermediate vocational school or secondary school; high = professional school or university.

^b ^Dutch version of the Center for Epidemiologic Studies Depression Scale [43].

^c ^Dutch version of the Hospital Anxiety and Depression Scale [47].

^d ^5-item Mastery Scale [48].

### Attrition

A total of 21% (50/244) of the sample did not complete the assessment at the end of 12 weeks (*t* *1*). We do not know the reasons for noncompletion of questionnaires. There were no significant differences between groups in completing *t* *1*. Nor were there significant differences between participants who did and who did not complete the *t* *1 *assessment (*P *< .10). This indicates that loss to follow-up was random. The assessment at 24 weeks (*t* *2*) was used to study the maintenance of effects in the MYM group and was not completed by 17% (21/121) of that group. There were several significant differences at baseline between the MYM participants who completed *t* *2 *and those who did not. Those who did not return that questionnaire were more likely to be male (χ^2^_1 _= 5.2, *P *= .02), to have lower education levels (χ^2^_2 _= 5.3, *P *= .07), and to have shown higher anxiety (*t*_119 _= 2.5, *P *= .01) and sense of mastery (*t*_119 _= −1.77, *P *= .08) at a previous assessment.

### Effects of the Intervention

Table 3 shows outcomes in the intention-to-treat sample for the primary (CES-D) and secondary (HADS Anxiety and Mastery) measures as produced by estimation-maximization imputation. The results of the regression analyses with adjustment for clustering were nearly identical to those without adjustment for clustering, indicating the absence of a cluster effect. We therefore present the results of independent-samples *t *tests. From baseline to 12 weeks, the MYM group showed significantly greater improvement in depressive symptoms, anxiety, and mastery than the control group, with a large effect size for depressive symptoms (CES-D, *d *= 0.94, 95% confidence interval [CI] 0.64–1.23) and moderate effect sizes for anxiety (HADS Anxiety, *d *= 0.49, 95% CI 0.24–0.75) and mastery (*d *= 0.44, 95% CI 0.19–0.70).

Table 4 shows the outcomes for the subsample completing the *t* *1 *questionnaires (with no imputation for the MYM or control group). These outcomes scarcely differed from those in Table 3, indicating that imputation had little effect on outcome. Table 5 shows outcomes for the subsample that attended at least one course session compared with the control group, using expectation-maximization imputation for missing values. Compared with the intention-to-treat sample in Table 3, effect sizes were slightly better still, with again a large effect for depressive symptoms (CES-D, *d *= 1.13, 95% CI 0.78–1.47) and moderate effects for anxiety (HADS Anxiety, *d *= 0.53, 95% CI 0.25–0.81) and mastery (*d *= 0.51, 95% CI 0.23–0.79).

**Table 3:** Effects of Master Your Mood (MYM) course: intention-to-treat analysis of full sample, expectation-maximization imputation.

Instrument	MYM group (n = 121)			Wait-listed controls (n = 123)			Between-group outcomes			
	Baseline (*t* *0*) mean (SD)	12 weeks (*t* *1*) mean (SD)	*d* ^a^	Baseline (*t* *0*) mean (SD)	12 weeks (*t* *1*) mean (SD)	*d*	Dif *d * ^b^	*t*	*df*	*P *value
Depression (CES-D)^c^	32.5 (8.4)	19.3 (9.7)	1.57	32.2 (8.2)	27.0 (8.6)	0.63	0.94	6.62	179.1	<.001
Anxiety (HADS)^d^	11.2 (3.6)	8.0 (3.9)	0.89	11.8 (3.7)	10.4 (3.3)	0.39	0.49	3.80	215.9	<.001
Mastery^e^	12.8 (3.4)	15.9 (4.1)	0.91	12.8 (3.6)	14.5 (3.5)	0.47	0.44	3.36	226.8	.001

^a ^Individual standardized effect size (*d *= *t* *0 *– *t* *1* /SD*t0*), with positive effect sizes indicating improvement.

^b ^Effect size differences between MYM group and wait-listed control group.

^c ^Dutch version of the Center for Epidemiologic Studies Depression Scale [43].

^d ^Dutch version of the Hospital Anxiety and Depression Scale [47].

^e ^5-item Mastery Scale [48].

**Table 4:** Effects of Master Your Mood (MYM) course: comparison of responding participants only, no imputation.

Instrument	MYM group (n = 96)			Wait-listed controls (n = 98)			Between-group outcomes			
	Baseline (*t* *0*) mean (SD)	12 weeks (*t* *1*) mean (SD)	*d* ^a^	Baseline (*t* *0*) mean (SD)	12 weeks (*t* *1*) mean (SD)	*d*	Dif *d * ^b^	*t*	*df*	*P *value
Depression (CES-D)^c^	32.5 (8.0)	19.1 (10.7)	1.60	31.6 (7.7)	26.7 (9.4)	0.60	0.99	5.69	179.1	<.001
Anxiety (HADS)^d^	10.9 (3.5)	7.8 (4.2)	0.88	11.7 (3.2)	10.2 (3.5)	0.39	0.49	3.10	192	.002
Mastery^e^	12.9 (3.3)	16.1 (4.3)	0.93	12.9 (3.3)	14.6 (3.8)	0.47	0.46	2.82	184.5	.005

^a ^Individual standardized effect size (*d *= *t* *0 *– *t* *1* /SD*t0*), with positive effect sizes indicating improvement.

^b ^Effect size differences between MYM group and wait-listed control group.

^c ^Dutch version of the Center for Epidemiologic Studies Depression Scale [43].

^d ^Dutch version of the Hospital Anxiety and Depression Scale [47].

^e ^5-item Mastery Scale [48].

**Table 5:** Effects of Master Your Mood (MYM) course: MYM participants attending at least one session compared with wait-listed control group, expectation-maximization imputation.

Instrument	MYM group (n = 96)			Wait-listed controls (n = 123)			Between-group outcomes			
	Baseline (*t* *0*) mean (SD)	12 weeks (*t* *1*) mean (SD)	*d* ^a^	Baseline (*t* *0*) mean (SD)	12 weeks (*t* *1*) mean (SD)	*d*	Dif *d * ^b^	*t*	*df*	*P *value
Depression (CES-D)^c^	33.2 (7.8)	19.6 (10.3)	1.76	32.2 (8.2)	27.0 (8.6)	0.63	1.13	6.15	170.6	<.001
Anxiety (HADS)^d^	11.3 (3.5)	8.6 (4.4)	0.92	11.8 (3.7)	10.4 (3.3)	0.39	0.53	3.53	217	.001
Mastery^e^	12.6 (3.4)	15.8 (4.1)	0.98	12.8 (3.6)	14.5 (3.5)	0.47	0.51	3.36	217	.001

^a ^Individual standardized effect size (*d *= *t* *0 *– *t* *1* /SD*t1*), with positive effect sizes indicating improvement.

^b ^Effect size differences between MYM group and wait-listed control group.

^c ^Dutch version of the Center for Epidemiologic Studies Depression Scale [43].

^d ^Dutch version of the Hospital Anxiety and Depression Scale [47].

^e ^5-item Mastery Scale [48].

### Reliable and Clinical Change

At 12 weeks, 56% (68/121) of the participants in the MYM group and 20% (24/123) in the control group showed reliable and clinically significant change (a positive change of 5 points or more on the CES-D in combination with a score below 22). This between-group difference was significant (χ^2^_1 _= 35.0, *P *< .001) and yielded a number needed to treat of 2.7.

### Maintenance of Effects in the MYM Group

Table 6 shows the results of the paired *t *tests for the MYM group (intention-to-treat sample). There was significant improvement in depressive symptoms, anxiety, and mastery from baseline (*t* *0*) to 12 weeks (*t* *1*), as well as from baseline to 24 weeks (*t* *2*). The effect sizes (*d*) were large for all measures. Sense of mastery even improved significantly with a small effect size from 12 weeks (*t* *1*) to 24 weeks (*t* *2*). The positive results achieved at 12 weeks were thus maintained at 24 weeks, and mastery continued improving.

**Table 6:** Effect maintenance in the Master Your Mood group (n = 121).

Instrument	Baseline (*t* *0*) Mean (SD)	12 weeks (*t* *1*) Mean (SD)	24 weeks (*t* *2*) Mean (SD)	*t* *0 * *− t* *1*			*t* *0 * *− t* *2*			*t* *1 * *− t* *2*		
				*t* *a*	*P *value	*d* *b*	*t*	*P *value	*d*	*t*	*P *value	*d*
Depression (CES-D)^c^	32.5 (8.4)	19.3 (9.7)	18.3 (10.7)	13.3	<.001	1.57	9.4	<.001	1.69	1.0	.31	0.10
Anxiety (HADS)^d^	11.2 (3.6)	8.0 (3.9)	7.8 (3.8)	12.8	<.001	0.89	10.0	<.001	0.96	0.8	.42	0.06
Mastery^e^	12.8 (3.4)	15.9 (4.1)	16.7 (4.1)	9.2	<.001	0.91	10.4	<.001	1.14	2.8	.006	0.19

^a^ *df *= 120.

^b ^Individual standardized effect sizes [*d *= (*t* *0* *− t* *1*) /SD*t0*); *d *= (*t* *0* *– t* *2*) /SD*t0 *; *d *= (*t* *1* *– t* *2*) /SD*t1*] with positive effect sizes indicating improvement.

^c ^Dutch version of the Center for Epidemiologic Studies Depression Scale [43].

^d ^Dutch version of the Hospital Anxiety and Depression Scale [47].

^e ^5-item Mastery Scale [48]. Higher scores stand for higher sense of mastery; differences calculated as *t* *1 − t* *0* *, t* *2* *− t* *0* *, t* *2* *− t* *1*.

### Sessions Attended and Outcome

Not all MYM group participants attended all course sessions: 21% (25/121) did not attend any sessions, 52% (63/121) attended at least four sessions, and 20% (24/121) attended all six. The average number of sessions attended was 3.2 (SD 2.2) with a range from 0 to 6.

Tested at *P *< .05, there were no significant differences in the CES-D mean effect sizes between those attending no sessions (*d *= 1.3) and those attending one or more (*d *= 1.6, *t*_119 _= 1.03, *P *= .31), nor between those attending fewer than 3 (*d *= 1.5) or more than 3 sessions (*d *= 1.6, *t*_110.6 _= 0.73, *P* *= *.47). Tested at *P *< .10, some differences emerged between participants attending no sessions and those attending at least one. Nonattendees included fewer experienced Web chatters (9/25, 36%) as compared with attendees (54/96, 56%; χ^2^_1 _= 3.3, *P *= .07), and nonattendees also had lower mean baseline CES-D scores (mean 29.6, SD 10, vs 33.2, SD 7.8; Wald χ^2^_1 _= 3.6, *P *= .06).

## Discussion

### Main Results

In this study, the Internet-based CBT group course known as Grip op Je Dip (Master Your Mood) for young people aged 16 to 25 years proved significantly more effective than a waiting-list control group in decreasing depressive symptoms. At 3 months, a large between-group effect size of 0.94 was found. The MYM group also showed a significantly greater reduction in anxiety symptoms (with a medium between-group effect of 0.49) and improvement in sense of control or mastery (medium effect of 0.44). The proportion of participants showing reliable and clinically significant change was 0.56 in the MYM group versus 0.20 in the control group (χ^2^_1 _= 35.0, *P *< .001). The reductions in depressive and anxiety symptoms and the increased sense of mastery were maintained in the MYM group at 6-month follow-up.

### Comparison With Other Work

Our study is one of the first randomized controlled trials to study online depression treatment for young people [32,33], and it is the first to focus on an online group course [30]. This hampers any solid comparison with prior Internet intervention research, which has chiefly focused on adults and on individual approaches. It is with this limitation in mind that we compare the results of the current study with other work.

The effectiveness of online treatment for depression in adults has already been demonstrated [24-26]. Our study showed the effectiveness of an online intervention for young people with depressive symptoms. The large effect size of 0.94 for the MYM course is comparable with the effects of face-to-face psychotherapy for depression in youth [52-54]. So far, effect size differences for Internet-based adult therapies for depression and anxiety have appeared to be related to the amounts of accompanying therapist support [55]. Since the MYM intervention provided substantial support by the course facilitator in the weekly group sessions, this might partly account for the large effect we obtained. In addition to the significant declines in CES-D scores, the MYM course was also associated with significantly lower scores on the HADS Anxiety subscale. Young people with primary anxiety may therefore also stand to benefit—a possibility supported in a recent trial that showed the effectiveness of an online CBT protocol for adult anxiety disorders and/or depression [56].

We also analyzed clinically significant change in the present study. The proportions of improved and recovered participants (56% in the MYM group vs 20% in the control group) seem in line with those for Web-based interventions for adult depression [57-59].

Dose–effect analysis in our study found no correlation between the number of sessions attended and the intervention outcomes, likewise corresponding to other studies [58-61]. Similarly, a meta-analysis by Weisz et al [62] found treatment duration not to be correlated with outcome. The explanation for this is not clear. In our study we did not ask the participants of the MYM group for their reasons for stopping after one or a few sessions. It could be that those who stopped felt they had recovered enough. This may have leveled out the correlation between sessions attended and outcome. The MYM group also included participants who did not attend any session, and they still displayed intervention effects. An explanation for this might be the difference in study conditions to which the MYM group and the wait-listed group were assigned: although both groups attended no sessions, the MYM participants made an active decision about this, while the wait-listed group did not. Indeed, which specific elements of a treatment are effective and which exact mechanisms bring on a person’s recovery are still at the frontiers of knowledge.

In the current field of Internet-based help, there is an ongoing search for an optimal balance (in terms of outcome and costs) between the relative amounts of therapist support and self-guidance [32]. In comparison with an individual eight-session CBT-based online self-help intervention with professional support [58], the facilitated six-session MYM course entailed less supervision time per participant (160 minutes in the eight-session CBT vs 135 minutes in six-session MYM, based on an average of 4 participants per facilitator), while the between-group effect size in the MYM was larger (0.94 MYM vs 0.72 CBT self-help at 12-week posttest). One possibility is that the group interaction, whereby group members provide some support to one another, might have boosted outcomes over the therapist support.

### Limitations

This study has several limitations. One of them is the infeasibility of comparing the 6-month (*t* *2*) outcomes of the MYM and control groups, since the control group had access to the course after *t* *1*. Another limitation is the passive (waiting-list) control condition. This makes it difficult to conclude that the treatment contained components that were “specifically effective over and above simple compassion, friendliness, attention and belief” [63]. Furthermore, the fact that participants were not blind to their condition, though generally inherent in psychotherapy studies, could have introduced some bias. The exclusive reliance on self-report measures is another limitation; other sources of information were missing. In this study we encountered missing data, though the attrition rate was much lower than in some other studies. High attrition rates are common in studies of Internet-based interventions [64,58]. We found no indication in the analyses that our missing data had affected the results. We dealt with missing values at posttest and follow-up using the expectation-maximization imputation method [49].

Our participants had rather high levels of education relative to the general population, so it is uncertain whether results can be generalized to people with lower education levels. The same can be said for male participants, who were underrepresented in our study in relation to the depression prevalence in the male general population. Adolescents aged 16 and 17 years were also underrepresented, due apparently not to lack of willingness to participate, but to a lack of consent given by parents. Further, we are uncertain whether results can be generalized to people with severe depression, as they were excluded from the study.

### Future Research Directions

We have pointed out that there is a lack of randomized controlled trials of Web-based interventions that specifically target adolescents and young adults with depression. From a preventive point of view, research on depression in youth is acutely needed, given the frequent early onset of subclinical and major depression and their far-reaching impacts. Future research should focus on the economic evaluation of Internet-based interventions for youth and on outcome research regarding stepped-care interventions (minimal where possible, sustainable where necessary). Trials are also needed in which online treatment groups are compared with active online control groups. This could help identify more specific elements of treatment that are effective. People with low socioeconomic status backgrounds are generally underrepresented in study samples but are in particular need due to their higher prevalence of psychological distress [3]. Interventions specially tailored to such groups therefore ought to be developed and studied.

### Conclusion

Despite the limitations of the present study, our findings suggest that adolescents and young adults with depressive symptoms can benefit from an Internet-based CBT group course. In our intervention, with a level of professional support per person roughly equivalent to that in other facilitated Web-based self-help interventions for depression, we found a relatively large effect size. The group aspect of our intervention may have accounted for this. Along with the individual approaches developed so far, online CBT group courses for depression may open additional opportunities to reach people who might otherwise remain untreated, thus achieving long-term cost savings. Future research should focus on economic evaluations of Internet-based interventions for depression in youth.

## Abbreviations

CBT: cognitive behavioral therapy
CES-D: Center for Epidemiologic Studies Depression Scale
CI: confidence interval
HADS: Hospital Anxiety and Depression Scale
MINI-Plus: Mini-International Neuropsychiatric Interview
MYM: Master Your Mood

## Multimedia Appendix 1

CONSORT EHEALTH V1.6 checklist [65].
