# Supplementary material for: Effectiveness of an Online Group Course for Depression in Adolescents and Young Adults: A Randomized Trial
Source: J Med Internet Res. 2012 Jun 7;14(3):e86. doi: 10.2196/jmir.2033 (PMC3414873; doi:10.2196/jmir.2033)
Supplement: Supplementary file 1 [file jmir_v14i3e86_app1.pdf]

CONSORT-EHEALTH Checklist V1.6 Report	**Manuscript Number**	2033
**Date completed**	8/25/2011 14:50:<br>29	
**by**	Rianne van der<br>Zanden	
Effectiveness of an online group course for depression in adolescents and young adults: a randomised trial		
TITLE		
**1a-i) Identify the mode of delivery in the title**		
Effectiveness of an "online group course" for depression in adolescents and young adults: a randomised trial		
**1a-ii) Non-web-based components or important co-interventions in title**		
not applicable		
**1a-iii) Primary condition or target group in the title**		
Effectiveness of an online group course "for depression in adolescents and young adults": a randomised trial		
ABSTRACT		
**1b-i) Key features/functionalities/components of the intervention and comparator in the METHODS section of the ABSTRACT**		
Methods<br>A total of 244 young people with depressive symptoms were randomised to the online MYM course or to a waiting-list control condition (WL).		
**1b-ii) Level of human involvement in the METHODS section of the ABSTRACT**		
This paper evaluates and discusses the effectiveness of the guided webbased group course called Grip op Je Dip (Master Your Mood- MYM), designed for young people aged 16 to 25 with depressive symptoms, in comparison with a wait-listed control group.		
**1b-iii) Open vs. closed, web-based (self-assessment) vs. face-to-face assessments in the METHODS section of the ABSTRACT**		
Participants were recruited from the general population by means of<br>promotional materials in general practitioners' offices and educational<br>institutions. Banners and links were also placed on mental health–related websites and on websites popular with young people.<br><br>Those interested were referred to the MYM website [39] to complete an<br>online preliminary screening questionnaire and apply for the course.		
**1b-iv) RESULTS section in abstract must contain use data**		
"A total of 244 young people with depressive symptoms were randomised to the online MYM course or to a waiting-list control condition (WL). The primary outcome measure was treatment outcome after three months on the Depression Scale (CES-D). Secondary outcomes were anxiety (HADS) and mastery (Mastery Scale)."<br><br><br>"The MYM group (n = 121) showed significantly greater improvement in depressive symptoms at 3 months than the<br>control group (n = 123) (t187 = 6.62, P < .001), with a large between-group effect size of d = 0.94 (95% confidence interval [CI]<br>0.64–1.23). The MYM group also showed greater improvement in anxiety (t187 = 3.80, P < .001, d = 0.49, 95% CI 0.24–0.75)<br>and mastery (t187 = 3.36, P = .001, d = 0.44, 95% CI 0.19–0.70)."		
**1b-v) CONCLUSIONS/DISCUSSION in abstract for negative trials**		
There were no negative results of the trial with regard to primary and<br>secondary outcome measures.		
INTRODUCTION		
**2a-i) Problem and the type of system/solution**		

"Given the high prevalence rates, the serious outcomes and the economic burden, the World Health Organisation calls for the development of preventive interventions to reduce the burden of this disorder [18] [19]. The first onset is usually in adolescence [20] and it is wise to intervene at an early stage. Yet young people experience many barriers to seeking professional help. They tend to deny or underestimate the problems, fear stigmatisation and question the benefits of help [21]. If they do seek help, they often encounter waiting lists [22] [23]."<br><br>"Internet-Based Interventions for Depression<br>By offering a solution to the stigmatization problem,<br>Internet-based approaches could help in reaching target groups<br>who might otherwise remain untreated. The Internet provides<br>anonymity and the opportunity to take part in an intervention<br>in the privacy of the home."		
**2a-ii) Scientific background, rationale: What is known about the (type of) system**		
"This study is one of the first RCTs to investigate online depression<br>treatment for young people, and the first to focus on an online group<br>course."		
METHODS		
**3a) CONSORT**		
"Objective<br>This paper evaluates and discusses the effectiveness of a web-based<br>group course called Grip op Je Dip (Master Your Mood), designed for<br>young people aged 16 to 25 with depressive symptoms, in comparison<br>with a wait-listed control group. The primary outcome measure is<br>depression, and secondary outcomes are anxiety and sense of control.<br>Based on the results of the pilot study [29] we expect better outcomes for the course group."		
**3b-i) Bug fixes, Downtimes, Content Changes**		
There were no changes to methods.		
**4a-i) Computer / Internet literacy**		
"Participants also had to be able to read, write and chat in Dutch on at<br>least primary school level."		
**4a-ii) Open vs. closed, web-based vs. face-to-face assessments:**		
"Participants were recruited from the general population by means of<br>promotional materials in general practitioners' offices and educational<br>institutions. Banners and links were also placed on mental health–related websites and on websites popular with young people.<br>Those interested were referred to the MYM website [39] to complete an<br>online preliminary screening questionnaire and apply for the course."		
**4a-iii) Information giving during recruitment**		
"Those interested were referred to the MYM website [39] to complete an online preliminary screening questionnaire and apply for the course.<br>Those with CES-D scores between 10 and 45 then received additional<br>information about the study, an informed consent form (including a<br>parental consent form for 16- and 17-year-olds) and a baseline<br>questionnaire. For course applicants scoring 25 to 45 on the CES-D, a<br>mandatory online chat session followed, in which suicidal ideation was<br>assessed with the MINI-Plus interview."		
**4b-i) Report if outcomes were (self-)assessed through online questionnaires**		
"The exclusive reliance on self-report measures is another limitation; other sources of information were missing."		
**4b-ii) Report how institutional affiliations are displayed**		
Not applicable.		
**5-i) Mention names, credential, affiliations of the developers, sponsors, and owners**		
"Declaration of interest<br>Rianne van der Zanden and Rob Gerrits are two of the developers of the online MYM group course, and the Trimbos Institute is a cooperation partner in MYM, but none of these derive financial income from the MYM intervention. There are no competing interests."		
**5-ii) Describe the history/development process**		

Objective<br>This paper evaluates and discusses the effectiveness of a web-based<br>group course called Grip op Je Dip (Master Your Mood), designed for<br>young people aged 16 to 25 with depressive symptoms, in comparison<br>with a wait-listed control group. The primary outcome measure is<br>depression, and secondary outcomes are anxiety and sense of control.<br>"Based on the results of the pilot study [29] we expect better outcomes for the course group."		
**5-iii) Revisions and updating**		
There were no changes in the application/intervention. I consider this as the regular situation during RCT's. Only when the intervention underwent changes, this has to be reported.		
**5-iv) Quality assurance methods**		
"Ethical approval was granted by an independent medical ethics<br>committee (CCMO no. NL18984.097.07). The trial is registered<br>(NTR1694), and the study protocol has been published [36].<br>Random allocation was automated by a computer program with no<br>interference by course facilitators or researchers. A blocked<br>randomisation scheme was used with blocks of two, stratified by<br>depressive symptoms (CES-D scores of 10-24 versus 25-45) and age...."		
**5-v) Ensure replicability by publishing the source code, and/or providing screenshots/screen-capture video, and/or providing flowcharts of the algorithms used**		
The manuscript contains a flow chart of participants, the URL of the MYM website, a textbox with the content of the intervention and a method section with a comprehensive descprition of all the essential elements for replicability.		
**5-vi) Digital preservation**		
The paper contains a detailed description of the intervention which is sufficient: see manuscript 'Method/conditions: the intervention'		
**5-vii) Access**		
See manuscript 'Recruitement procedure'<br><br>"Participants were recruited from the general population by<br>means of promotional materials in general practitioners' offices<br>and educational institutions. Banners and links were also placed<br>on mental health-related websites and on websites popular with<br>young people. There were no explicit restrictions on country of<br>origin, but the course language was Dutch and the recruitment<br>took place in Dutch."		
**5-viii) Mode of delivery, features/functionalities/components of the intervention and comparator, and the theoretical framework**		
see manuscript 'Method/conditions: the intervention':<br><br>"Conditions: the intervention<br>The online MYM course is based on the face-to-face intervention of the<br>same name, which was developed by the Trimbos Institute, the<br>Netherlands Institute of Mental Health and Addiction. That intervention<br>was derived from the Dutch version [40] of the Coping with Depression<br>course [41]. The face-to-face course was adapted to the Internet in a..."		
**5-ix) Describe use parameters**		
"The course we evaluated took place at fixed times in a secured chatroom, which participants entered with their username and password. Anonymity within the group was ensured by a self-chosen nickname. SMS reminders were sent to participants' mobile phones one-half hour before each session. The course comprised six sessions of 90 minutes each, each at a set time every week, and home exercises. The sessions were structured around six themes (see box 1). During the sessions, course material was introduced by the facilitators and displayed in the chatroom using text and.."		
**5-x) Clarify the level of human involvement**		

"The course was guided by one or two trained professionals, depending on group size (6 participants was the maximum).<br>During the sessions, course material was introduced by the facilitators<br>and displayed in the chatroom using text and images. Participants could<br>respond, share experiences and ask questions.<br>Eligible applicants were randomised to the intervention group (MYM) or..."		
**5-xi) Report any prompts/reminders used**		
"Applicants were informed of their allocation by e-mail and received a<br>tailored referral if declined. Participants then received a personal e-mail<br>from their facilitator to inform them of the specific times and dates of the course, the homework assignment for the first session, and a username and password for the chatroom."<br><br>"Anonymity within the group was ensured by a self-chosen nickname. SMS reminders were sent to participants' mobile phones one-half hour before..."		
**5-xii) Describe any co-interventions (incl. training/support)**		
"Participating mental health agencies<br>A total of 14 mental health care agencies participated in the project, all<br>working with online course participants from all over the Netherlands. The courses were supervised by professional mental health promotion<br>workers, trained in administering the MINI-Plus interview and in<br>conducting the course."		
**6a-i) Online questionnaires: describe if they were validated for online use and apply CHERRIES items to describe how the questionnaires were designed/deployed**		
"Depressive symptoms. The Center for Epidemiologic Studies Depression Scale (CES-D) [43] [44] measures the frequency of 20 depressive symptoms over the past week on a 4-point Likert scale. The total score may range from 0 to 60, with higher scores indicating higher levels of depression. Computerised and paper-and-pencil versions of the CES-D correlate at a very high level [45]. The web-based version of the CES-D has been shown to be a reliable and valid screening instrument in a Dutch adolescent population, with a Cronbach's alpha of 0.93 [46]."		
**6a-ii) Describe whether and how "use" (including intensity of use/dosage) was defined/measured/monitored**		
The 'use' was defined as the amount of sessions the participant attended.<br><br>"Sessions attended and outcome<br>Not all MYM group participants attended all course sessions: 20.7%<br>(25/121) did not attend any sessions; 52.1% (63/121) attended at least<br>four sessions and 19.8% (24/121) attended all six. The average number...."<br>of sessions attended was 3.2 (SD=2.2) with a range from 0 to 6.		
**6a-iii) Describe whether, how, and when qualitative feedback from participants was obtained**		
No qualitative feedback from participants was obtained.		
**7a-i) Describe whether and how expected attrition was taken into account when calculating the sample size**		
See the Reply document:<br>COMMENT 8. Was the clustering of centres accounted for in your sample<br>size calculations or potential drop-out?<br>REPLY 8. No, this was originally not the case. We would have needed a<br>larger sample to detect the effect size we were using in the power<br>calculation. However, the effect found was larger than expected and<br>therefore we had enough power to detect those effects as significant.		
**7b) CONSORT**		
Not applicable.		
**8a) CONSORT**		
Not applicabale; the care providers were not allocated to a particulair trial group.		
**8b) CONSORT**		

"Eligible applicants were randomised to the intervention group (MYM) or<br>the control group (WL, wait-listed for 14 weeks). Random allocation was<br>automated by a computer program with no interference by course<br>facilitators or researchers. A blocked randomisation scheme was used<br>with blocks of two, stratified by depressive symptoms (CES-D scores of 10-24 versus 25-45) and age (younger versus older than 18). The<br>outcome of the randomisation was generated and made available at the<br>moment the course facilitator indicated that the applicant was eligible for....."		
**9) CONSORT**		
"Eligible applicants were randomised to the intervention group (MYM) or<br>the control group (WL, wait-listed for 14 weeks). Random allocation was<br>automated by a computer program with no interference by course<br>facilitators or researchers. A blocked randomisation scheme was used<br>with blocks of two, stratified by depressive symptoms (CES-D scores of 10-24 versus 25-45) and age (younger versus older than 18). The<br>outcome of the randomisation was generated and made available at the<br>moment the course facilitator indicated that the applicant was eligible for..."		
**10) CONSORT**		
Eligible applicants were randomised to the intervention group (MYM) or<br>the control group (WL, wait-listed for 14 weeks). "Random allocation was automated by a computer program with no interference by course<br>facilitators or researchers. A blocked randomisation scheme was used<br>with blocks of two, stratified by depressive symptoms (CES-D scores of 10-24 versus 25-45) and age (younger versus older than 18). The<br>outcome of the randomisation was generated and made available at the<br>moment the course facilitator indicated that the applicant was eligible for...."		
**11a-i) Specify who was blinded, and who wasn't**		
"The total research procedure was automated. There were no outcome<br>assessors. The row data were deliverd in a 'vsv file' by an external<br>webmaster and conversed into spss-files by the researcher. The<br>participants were not blind to their condition."<br><br>see manuscript Limitations:<br>"Furthermore, the fact that participants were not blind to their condition, though generally inherent in psychotherapy studies, could have<br>introduced some bias."		
**11a-ii) Discuss e.g., whether participants knew which intervention was the "intervention of interest" and which one was the "comparator"**		
see manuscript Limitations:<br>"Furthermore, the fact that participants were not blind to their condition,<br>though generally inherent in psychotherapy studies, could have<br>introduced some bias."		
**11b) CONSORT**		
Not applicable.		
**12a) CONSORT**		
Not applicable.		
**12a-i) Imputation techniques to deal with attrition / missing values**		
"The expectation-maximisation (EM) method was used to impute data<br>missing at t1 and t2. It imputes values by maximum-likelihood estimation using the observed data in an iterative process [50]. These analyses were<br>based on the intention-to-treat principle, including data from all participants, whether or not they received the intervention."		
**12b) CONSORT**		
"The proportion of participants showing reliable and clinically significant<br>improvement [52] was determined in terms of an improvement of 5 points on the CES-D in combination with a score lower than 22 on the CES-D (cut-off based on Cuijpers et al. [46]). Subsequently, the number needed to treat (NNT) was calculated as 1/risk difference. The NNT indicates here how<br>many young people with depressive symptoms would need to take the MYM course in order to generate a health gain in one person over 12 weeks."		
RESULTS		

**13a) CONSORT**		
"Recruitment took place from 20 May 2008 to 6 March 2010. Figure 1<br>shows the flow of participants through the trial. Of the 974 people who<br>applied for the online MYM course, 244 (25.1%) were included in the<br>study. Reasons for non-inclusion were lack of informed consent (30.0%, 219/730), CES-D depression score outside the 10-45 range (27.7%, 202/730), no-show at the MINI-Plus interview (24.9%, 182/730), and age outside the 16-25 range (12.7%, 93/730). Additional exclusions were made for suicidal ideation (4.2%; 31/730), and other reasons......"		
**13b) CONSORT**		
see flow diagram attached to the manuscript. (Figure 1)		
**13b-i) Attrition diagram**		
"Attrition<br>A total of 20.5% (50/144) of the sample did not complete the assessment at the end of 12 weeks (t1). Reasons for non-completion of questionnaires are unknown. There were no significant differences between groups in completing t1. Nor were there significant differences between participants who did and who did not complete the t1 assessment (p < .10). This indicates that loss to follow-up was random. The assessment at 24 weeks....."		
**14a) CONSORT**		
"Recruitment took place from 20 May 2008 to 6 March 2010. Figure 1<br>shows the flow of participants through the trial."		
**14a-i) Indicate if critical “secular events” fell into the study period**		
Not applicable. Only when this is the case it should be reported.		
**14b) CONSORT**		
not applicable.		
**15) CONSORT**		
see manuscript, table 2.		
**15-i) Report demographics associated with digital divide issues**		
see manuscript, Table 2. Baseline characteristics of the 244 participants.		
**16-i) Report multiple “denominators” and provide definitions**		
see manuscript Results:"<br><br>"Effects of the intervention<br>Table 3 shows outcomes in the intention-to-treat sample for the<br>primary (CES-D) and secondary (HADS Anxiety and Mastery)<br>measures as produced by estimation-maximization imputation."<br><br>"Table 3. Effects of Master Your Mood (MYM) course: intention-to-treat analysis of full sample, expectation-maximization imputation.		
**16-ii) Primary analysis should be intent-to-treat**		
see manuscript Results, table 3,4,5		
**17a) CONSORT**		
"The MYM group (n = 121) showed significantly greater improvement on depressive symptoms at three months than the WL-group (n = 123) (t(187) = 6.62, p < .001, 95% CI = 0.64-1.23), with a large between-group effect size of d = 0.94. The MYM group also showed greater improvement on anxiety (t (187) = 3.80, p < .001, d = 0.49, 95% CI = 0.24-0.75) and mastery (t(187) = 3.36, p = .001, d = 0.44, 95% CI = 0.19-0.70). At 12 weeks, 56.2% (68/121) of the participants in the MYM group and 19.5 % (24/123) in the WL group showed reliable and clinically significant change."		
**17a-i) Presentation of process outcomes such as metrics of use and intensity of use**		

"Sessions Attended and Outcome<br>Not all MYM group participants attended all course sessions:<br>21% (25/121) did not attend any sessions, 52% (63/121)<br>attended at least four sessions, and 20% (24/121) attended all<br>six. The average number of sessions attended was 3.2 (SD 2.2)<br>with a range from 0 to 6. Tested at $P < .05$, there were no significant differences in the CES-D mean effect sizes between those attending no sessions<br>(d = 1.3) and those attending one or more (d = 1.6, t_{119} = 1.03,P = .31), nor between those attending fewer than 3 (d = 1.5) or<br>more than 3 sessions (d = 1.6, $t_{110.6}$ = 0.73, P = .47). Tested at<br>$P < .10$, some differences emerged between participants<br>attending no sessions and those attending at least one.<br>Nonattendees included fewer experienced Web chatters (9/25,<br>36%) as compared with attendees (54/96, 56%; χ^2<br>1 = 3.3, P =.07), and nonattendees also had lower mean baseline CES-D scores (mean 29.6, SD 10, vs 33.2, SD 7.8; Wald χ^2 1 = 3.6, P= .06)."		
**17b) CONSORT**		
Not applicable.		
**18) CONSORT**		
"Reliable and clinical change<br>At 12 weeks, 56.2% (68/121) of the participants in the MYM group and<br>19.5 % (24/123) in the WL group showed reliable and clinically significant change (a positive change of 5 points or more on the CES-D in combination<br>with a score below 22). This between-group difference was significant (2(1) = 35.0, $p < .001$) and yielded an NNT of 2.7."		
**18-i) Subgroup analysis of comparing only users**		
"Tested at $P < .05$, there were no significant differences in the<br>CES-D mean effect sizes between those attending no sessions<br>(d = 1.3) and those attending one or more (d = 1.6, t_{119} = 1.03,<br>P = .31), nor between those attending fewer than 3 (d = 1.5) or<br>more than 3 sessions (d = 1.6, $t_{110.6}$ = 0.73, P = .47). Tested at<br>$P < .10$, some differences emerged between participants<br>attending no sessions and those attending at least one.<br>Nonattendees included fewer experienced Web chatters (9/25,<br>36%) as compared with attendees (54/96, 56%; χ^2<br>1 = 3.3, P = .07), and nonattendees also had lower mean baseline CES-D scores (mean 29.6, SD 10, vs 33.2, SD 7.8; Wald χ^2<br>1 = 3.6, P = .06)."		
**19) CONSORT**		
not apllicable.		
**19-i) Include privacy breaches, technical problems**		
Not applicable; there were no problems of this kinds.<br>When this was the case, this should be reported.		
**19-ii) Include qualitative feedback from participants or observations from staff/researchers**		
This kind of information is presented in another publication, by Gerrits et<br>al., 2007. (see 'references' manuscript)		
DISCUSSION		
**20-i) Typical limitations in ehealth trials**		

"Limitations<br>This study has several limitations. One of them is the infeasibility of<br>comparing the six-month (t2) outcomes of the MYM and control groups,<br>since the latter had access to the course after t1. Another limitation is the passive (waiting list) control condition. This makes it difficult to concludethat the treatment contains components that are ‘specifically effective over and above simple compassion, friendliness, attention and belief’ [64].<br>Furthermore, the fact<br>that participants were not blind to their condition, though<br>generally inherent in psychotherapy studies, could have<br>introduced some bias. The exclusive reliance on self-report<br>measures is another limitation; other sources of information<br>were missing. In this study we encountered missing data, though<br>the attrition rate was much lower than in some other studies.<br>High attrition rates are common in studies of Internet-based<br>interventions [64,58]. We found no indication in the analyses<br>that our missing data had affected the results. We dealt with<br>missing values at posttest and follow-up using the<br>expectation-maximization imputation method [49]."		
**21-i) Generalizability to other populations**		
"Our participants had rather high levels of education relative to the general population, so it is uncertain whether results can be generalised to people with lower education levels. The same can be said for male participants, who were underrepresented in our study in relation to the depression prevalence in the male general population. Adolescents aged 16 and 17 were also underrepresented, due apparently not to lack of willingness to participate, but to a lack of consent given by parents. Further, it is uncertain whether results can be generalised to people with severe depression, as they were excluded from the study."		
**21-ii) Discuss if there were elements in the RCT that would be different in a routine application setting**		
"The MYM group also included participants who did not attend any<br>session, and they still displayed intervention effects. An explanation for<br>this might be the difference in study conditions to which the MYM group<br>and the wait-listed group were assigned: although both groups attended<br>no sessions, the MYM participants made an active decision about this,<br>while the wait-listed group did not. Fact is, which specific elements of a<br>treatment are effective and which exact mechanisms bring on a person’s recovery are still at the frontiers of knowledge."		
**22-i) Restate study questions and summarize the answers suggested by the data, starting with primary outcomes and process outcomes (use)**		
"Main Results<br>In this study, the Internet-based CBT group course known as<br>Grip op Je Dip (Master Your Mood) for young people aged 16<br>to 25 years proved significantly more effective than a<br>waiting-list control group in decreasing depressive symptoms.<br>At 3 months, a large between-group effect size of 0.94 was<br>found. The MYM group also showed a significantly greater<br>reduction in anxiety symptoms (with a medium between-group effect of 0.49) and improvement in sense of control or mastery<br>(medium effect of 0.44). The proportion of participants showing<br>reliable and clinically significant change was 0.56 in the MYM<br>group versus 0.20 in the control group ($\chi^2_1 = 35.0$, $P < .001$).<br>The reductions in depressive and anxiety symptoms and the<br>increased sense of mastery were maintained in the MYM group<br>at 6-month follow-up."		
**22-ii) Highlight unanswered new questions, suggest future research**		

"Future Research Directions<br>We have pointed out that there is a lack of randomized<br>controlled trials of Web-based interventions that specifically<br>target adolescents and young adults with depression. From a<br>preventive point of view, research on depression in youth is<br>acutely needed, given the frequent early onset of subclinical<br>and major depression and their far-reaching impacts. Future<br>research should focus on the economic evaluation of<br>Internet-based interventions for youth and on outcome research<br>regarding stepped-care interventions (minimal where possible,sustainable where necessary). Trials are also needed in which<br>online treatment groups are compared with active online control<br>groups. This could help identify more specific elements of<br>treatment that are effective. People with low socioeconomic<br>status backgrounds are generally underrepresented in study<br>samples but are in particular need due to their higher prevalence<br>of psychological distress [3]. Interventions specially tailored to<br>such groups therefore ought to be developed and studied."		
Other information		
**23) CONSORT**		
Trial Registration: NTR1694<br>URL: http://www.trialregister.nl/trialreg/admin/rctview.asp?TC=1694		
**24) CONSORT**		
"Trial Registration: NTR1694<br>URL: http://www.trialregister.nl/trialreg/admin/rctview.asp?TC=1694<br>The trial is registered (NTR1694), and the study protocol has been<br>published [36]."		
**25) CONSORT**		
"Financial disclosure<br>The study was funded by a research grant from ZonMw (Netherlands<br>Organisation for Health Research and Development), grant no. 61300036.<br>No funding bodies had any role in study design, data collection and<br>analysis, decision to publish, or preparation of the manuscript."		
**X26-i) Comment on ethics committee approval**		
"Ethical approval was granted by an independent medical ethics<br>committee (CCMO no. NL18984.097.07). The trial is registered<br>(NTR1694), and the study protocol has been published [36]."		
**x26-ii) Outline informed consent procedures**		
"Those with CES-D scores between 10 and 45 then received additional<br>information about the study, an informed consent form (including a<br>parental consent form for 16- and 17-year-olds) and a baseline<br>questionnaire."		
**X26-iii) Safety and security procedures**		
"Ethical approval was granted by an independent medical ethics<br>committee (CCMO no. NL18984.097.07)."		
**X27-i) State the relation of the study team towards the system being evaluated**		
"Declaration of interest<br>Rianne van der Zanden and Rob Gerrits are two of the developers of the online MYM group course, and the Trimbos Institute is a cooperation partner in<br>MYM, but none of these derive financial income from the MYM intervention. There are no competing interests."		
